# Experience of the Absence of the Journey to Sessions in Clients' Narratives About Online Psychotherapy

**DOI:** 10.3389/fpsyg.2022.798960

**Published:** 2022-02-16

**Authors:** Dariusz Galasiński, Justyna Ziółkowska, Magdalena Witkowicz

**Affiliations:** ^1^Centre for Interdisciplinary Research into Health and Illness, University of Wroclaw, Wrocław, Poland; ^2^Faculty of Psychology, SWPS University of Social Sciences and Humanities, Wrocław, Poland

**Keywords:** discourse analysis, qualitative, psychotherapy, journey, online psychotherapy

## Abstract

**Background:**

Remotely provided psychotherapy due to the COVID-19 pandemic became common. One of the most significant changes related to providing online psychotherapy services is that clients no longer travel to their sessions.

**Aims:**

In the article we are interested in the narrated experience of the absence of journey to psychotherapy sessions. We study clients' stories of past journeys and how their absence, resulting from the change of the mode of therapy provision, is coped with and replaced by other activities in their narratives.

**Methods:**

The study takes a constructionist approach to discourse and focuses on the lexico-grammatical form of the notes. The data come from 12 semi-structured interviews with people who declared attending remote psychotherapy sessions after the national lockdown had been introduced.

**Results:**

In the collected data, the physical journey is constructed not only as travel time, solitude which can be used for reflection, but, importantly, as an active process which ends with a resolution. In contrast, in narratives of the time before an online session, constructions of unfettered agents are replaced with those of people whose actions are hedged and qualified.

**Conclusion:**

We argue that in the informants' narratives the journey to psychotherapy is meaningful and is part of the therapeutic process. We propose that it is a time of passing between two states—one before therapy and one in session. The journey therefore is experienced as a process of change, and not only a process of traveling.

## Introduction

The first attempts to deliver psychotherapy *via* teletechnologies date back to the 1960s (Wittson et al., 1961). Over the last two decades, many professionals implemented these methods as promising, cost-effective and helpful means to bridge the gap between rural areas and cities due to uneven distribution of mental healthcare services (Norman, 2006; Berryhill et al., 2018). However, some psychotherapists considered remote interventions lacking in interpersonal richness and physical expression (Norwood et al., 2018). There were also concerns about confidentiality, security and managing of emergencies (Glueckauf et al., 2018). According to the study published in 2018, 52% of psychologists indicated that none of their ongoing practice was provided online. Only a little over a quarter of respondents reported using videoconferencing technology for counseling in the year prior to the survey (Glueckauf et al., 2018).

The interest in online psychotherapy shifted rapidly in 2020. Since the outbreak of the COVID-19 pandemic, the use of remotely provided health services, including psychotherapy, has been skyrocketing (Jobes et al., 2020). Results of online surveys conducted among psychotherapists in 2020 show that the number of patients treated weekly *via* internet increased by 6,558% in Germany, 1,561% in Austria, 1,200% in Czech Republic and 343% in Slovakia. The effect for telephone psychotherapy was also substantial, with 213% increase in Germany, 979% in Austria, 417% in Czech Republic and 187% in Slovakia (Humer et al., 2020). Although no comparable Polish data are available at the moment of writing this article, it is reasonable to assume unprecedented increase in the practice of remote psychotherapy as well.

As psychotherapists and their patients had to stay physically apart, many of them were forced to try tele-mental health for the first time (Chen et al., 2020). Prior to the pandemic, these interventions were seldom used to treat serious mental illnesses (Miu et al., 2020). Over the last 2 years telepsychotherapy has been implemented to address conditions as severe as psychosis (Kopelovich and Turkington, 2021), bipolar disorder, depression and post-traumatic stress disorder (Miu et al., 2020). Remote interventions were also created for patients with a high risk of suicide (Jobes et al., 2020). In addition, the need for remotely provided mental healthcare has become more apparent due to the psychological impact of COVID-19 (Campbell, 2020; Wang et al., 2020).

One of the most significant changes related to providing online psychotherapy services is that clients no longer travel to their sessions. The existing research into traveling to psychotherapy sessions focuses mainly on the benefits of remote access and its advantages over traditional, face-to-face consultations. In most studies, the journey is presented as a burden, as it implies travel costs and time waste. Indeed, researchers suggest significant savings for clients (Ashwick et al., 2019; Khanra et al., 2021). Moreover, researchers indicate (Fleuty and Almond, 2020; Botta et al., 2021) that teletherapy is easier to fit in clients' busy schedules of patients, while at the same time clients gain control over the venue where sessions take place, which for some clients can be very important (Chen et al., 2020; Fleuty and Almond, 2020; Botta et al., 2021). Patients receiving telepsychiatry care during the pandemic also consider not having to commute to the clinic beneficial due to the saved cost and time, as they report in an online survey (Guinart et al., 2020). However, none of the studies discussed above examine the journeys and then the subsequent lack of them in depth.

There is a significant gap in the research on experience of the journey to or from psychotherapy sessions. While some clients enjoy the flexibility of remote meetings, others must rely on their limited resources (e.g., accommodation and computing) and find it harder to focus, as they get distracted by the household members (Russell, 2018; Ashwick et al., 2019; Biancalani et al., 2021). In interviews with military veterans suffering from PTSD, the participants mentioned struggling with the immediate comeback to reality after Skype sessions, while they needed time to reflect on them (Turgoose et al., 2018). One of the ways to cope with it was going for a walk (Turgoose et al., 2018). Researchers suggest that traveling to psychotherapy can be a part of the commitment to therapy, as patients note that online sessions can be canceled at a moment's notice (Ashwick et al., 2019; Fleuty and Almond, 2020). The data referred to in this paragraph are derived from qualitative studies on attitudes toward remote therapy. There is no research study that we are aware of dedicated to exploring the meaning of journeys and the rituals that could replace them.

Summing up, travel time to psychotherapy sessions is mostly described as an inconvenience that makes psychotherapy more difficult, expensive and less user-friendly (e.g., Khazaie et al., 2016). In contrast, in our article, we want to look at it from the clients' perspective, anchoring it within the therapeutic process. Even though, the key part of the process is what happens in the office, i.e., actions, experiences, and the relationship between the patient and the therapist when they are physically together, the role of events outside therapy sessions is also noted in the literature (Orlinsky et al., 2004). In our exploratory study we show that in narratives of some psychotherapy clients the journey to sessions is constructed as a special time. On this basis, we argue that the journey to psychotherapy can be seen as a potentially important element of the therapeutic process and not only as inconvenience. More research is needed into the role of the physical journey and, crucially, whether the journey can be replaced at times when it cannot be made.

## Aims and Methodology

In this article we are interested in the narrated experience of the absence of the journey to psychotherapy sessions. We explore clients' stories of past journeys and their absence. We are also interested in how, in the informants' narratives, the absence of the journey resulting from the change of the mode of therapy provision is coped with and replaced by other activities. In a nutshell, we argue here that the journey to psychotherapy should be viewed and engaged with as a meaningful activity and time which helps at least some clients in getting more from their therapists.

### The Data

The data come from an exploratory qualitative project on experience of the change in the communication channel with a psychotherapist. It was carried out during the SARS-Cov-2 pandemic lockdown in Poland, which was introduced on 12 March 2020 and made stricter on 25 March, limiting non-family gatherings to two people and religious gatherings to six and forbidding non-essential travel. The lockdown measures required psychotherapists to suspend face-to-face sessions and move to offering remote service *via* audio or video-audio channels.

The data were collected between 10 May and 24 May 24, 2020, ~8–10 weeks after the beginning of the lockdown. Participation in the research was voluntary, and no incentives were provided. The project was advertised on three internet fora for people using psychotherapeutic services. Twelve persons contacted the researcher and declared attending remoted psychotherapy sessions after the national lockdown had been introduced.

Our informants were 11 females and one male. All participants spoke fluent, native-like Polish, all had Polish sounding names, while the recordings do not suggest any non-standard language characteristics. They all used the Internet in Polish. The mean age of the females was 32 years (range: 24–47); the male was 32 years old. The mean duration of using psychotherapy services before the lockdown was 12 months and ranged from 2 weeks to 22 months. Four persons talked with the therapist on the phone and the remaining 8 *via* Internet communicators (Skype, Zoom, WhatsApp). The therapies, in various paradigms, were both on the national health service (3 persons) and attended privately (9 persons).

We used semi-structured interviews to explore: (1) experience of the change of the channel of communication; (2) circumstances of the first remote session; (3) consequences of the channel change. All interviews were conducted by a person who was trained in qualitative research interviews and had had no previous relationships with the informants. The interviews were conducted online *via* the Google Meet communicator. All participants provided written informed consent for participation in the study and being interviewed. The interviews were audio-recorded and subsequently transcribed verbatim.

All personal and other information which could lead to revealing the identity of the informants was removed. All the interviews were transcribed and the analyses were based on the original Polish data. For the purpose of this article, the fragments we quote were separately translated by two researchers from the Polish originals and then compared for accuracy and consistency. The translation is as close as possible to the original Polish version, aiming not only to render what was said, but also how it was said. That occasionally results in “bad” or disjointed English.

### Methodology

The analytic process consisted of two stages. The goal of the first stage was identification of fragments of narratives referring to the aims of the study and through this creating a separate subset of the data. Following, Potter and Wetherell (1987) we adopted selective coding as a means of reducing the volume of the data for a more intensive analysis of the narratives. The data were coded with the use of the MaxQDA software.

The first stage started with immersing ourselves in the data, which involves the repeated reading of the material (Braun and Clarke, 2006). Next, two authors (JZ and DG) started the coding process as a method for reducing the analytic material (Miles and Huberman, 1994). The process started with regular discussions of the data and the code system. As a unit of coding we adopted a syntactically determined proposition unit that referred to the physical journey to the therapist's office or its absence and to preparations for online sessions after the change to the online mode.

After creating the code system, the two authors separately coded the transcribed interviews The coded entries were subsequently compared and the differences were discussed and resolved (see Kvale and Brinkmann, 2009). On this basis the final dataset for detailed discourse analysis was created.

A text-oriented version of Critical Discourse Analysis (CDA) was used in the second, discourse analytic stage of data analysis. The assumption underlying this method is that social reality is constructed through and within language and that language is designed to represent reality selectively imbued with decisions about how to arrange them. Each of these selections carries its share of implicit assumptions, so that the reality represented is ideologically constructed (Hodge and Kress, 1993). It is also through discourse (i.e., practices of representation) that language users constitute social realities: their knowledge of social situations, the roles they play, their identities and relations with other social groups (Van Leeuwen and Wodak, 1999). No text, spoken or written, represents reality in a neutral or objective way, representation is never of reality “as it really is”, rather reality is always viewed through the tinted lens of ideological assumptions (Fairclough, 1992; Barker and Galasiński, 2001).

In the case in qualitative research, the theoretical approach (CDS) is also a method of analysis. And so, there is no protocol in CDS or a clearly defined method of analysis. In our study, we took a text-oriented approach (Fairclough, 1992, 1995, 2003). We were interested in the form of stretches of discourse, with an interest both in the semantics and syntax of the text, as well as the functions of what was said within the local context, and the social actions thus accomplished. We are particularly indebted to the developments of Halliday (1978); Halliday's (1994) and Halliday and Hasan's (1985) functional linguistics, with its main proposition that the analysis of lexico-grammatical form of language should be foregrounded as a resource for constructing meaning (Halliday, 1994). Elements of grammar and lexis were analyzed predominantly as having a particular function when used by speakers. In what follows, we focus primarily upon the ideational function of what the informants said, that is to say, we were predominantly interested in how they represented extralinguistic reality. But we also focused on the content of what is written, relating it to the larger socio-political context in which it is used. Using both the systemic-linguistic analysis (Halliday, 1994), as well as a hermeneutic-like interpretation of discourses, in terms of the context in which they were submerged, we attempt to reach the ideological underpinnings of the participants' experiences (Titscher et al., 2000).

We are therefore interested in the discourses our participants draw upon when they spoke during their interviews. Language users are not isolated individuals, but they are engaged in communicative activities as members of social groups, organizations, institutions, cultures, in the present analysis: clients of psychotherapy services whose psychotherapy was moved to a remote mode of contact. We want to discover parts of the “discourses of the physical journey to psychotherapy”, ways in which the concept was made social through the process of narrating it. in the presentation of the results of our analysis, we present the fragments which were agreed by the authors to fully represent the data at hand.

Importantly, as qualitative researchers we bracket off the issue of representativeness of the dataset. We do not wish to make claims as to the extent the study is representative of psychotherapy clients in a similar situation. Rather, we were interested in uncovering the discourse underpinning the journey to psychotherapy sessions and shed light upon it. We do realize that our data were contextual and cannot be thought of in terms of universality. It is, however, worth bearing in mind that language users can, but rarely do control the linguistic form of what they say or write. In such a way focusing upon the form of what our correspondents said we begin to explore what we earlier tentatively called “discourses of journey to psychotherapy”.

## The Results

### The Journey

As we expected based on previous research (Russell, 2018; Ashwick et al., 2019), the journey to psychotherapy sessions was represented in the narratives as the time devoted to reflecting on the previous and upcoming sessions. The shift from face-to-face sessions to those online was represented as missing the journey time and opportunity for the passage to therapy time. What we also found, however, was that the journey was consistently constructed in active terms, with the clients representing themselves as actively claiming the time for themselves.

Consider the following examples:

Extract 1. and in the same way, like, I think that when I was physically going to classes, to the university, I was going out, getting dressed, going to the (bus) stop, then it was a form of, like, I don't know, preparing, thinking about it.
Extract 2. and so always before the appointment there was this time and this click in my head that, okay, I'm going to the session, I'm concentrating on myself now, I'm focusing on myself now.
Extract 3. and I miss this traveling bit as I always dedicate this journey to the (therapist's) office to sort things out in my head.
Extract 4. I mean, I actually miss this travel time, when I sorted everything out in my head. It's not like I was thinking about what I wanted to say only during the journey, 'cause I was thinking about it all week, but the journey was the kind of moment when I was sorting everything out in my head. And I knew what was the most important and how, and I guess it was also some kind of mental preparation as well. And now I miss it a lot.

In the extracts, the speakers construct themselves as agents doing things during the journey to their psychotherapy sessions. The first two examples focus more on the physical journey, the others more on the mental activity. The first three extracts are the most explicit in representing the speakers as unfettered agents. The speaker in extract 1 positions herself as taking a series of actions, representing them in material processes through verbs of doing (Halliday, 1994). She speaks of going, leaving, getting dressed, walking. Similarly in Extract 2, the speaker shows herself as taking the route. Here, however, she also adds two mental processes (Halliday, 1994)—she speaks of concentrating and focusing on herself. They also position the speaker as an agent.

Extract 3 is slightly different as the journey is represents as a gerund (traveling), but the mental action of devoting is linked to ordering things in the head, also positions the speaker as a doer. Such constructions are similar to extract 4 where the speaker constructs her agency through reference to her mental activities.

What is crucial in these extracts is that all of them represent the speakers as active in the journey to their psychotherapy sessions. Whether their agency is represented through material process (verbs of doing) or mental processes (verbs referring to thinking), they all represent the journey not just as happening. The journey to psychotherapy is represented as one which is incidental, something that results from the venue where, linguistically, it is a deliberate action on the part of the client.

It is worth adding that Extract 4 represents the journey not so much in terms of the travel, but as time during which the speaker could engage in reflection. Moreover, the end of the fragment (“I knew what was the most important”) suggests an end to the thinking and “putting things in order”. The journey, it seems, does not end in the session, it ends in a resolution to the thinking process.

Also in the following two extracts, the speakers not only suggest that the journey is a useful time for thinking, but also imply that it results in decisions or “order”.

Extract 5. And even the way to the office was this kind of moment to think everything through one more time, to be with myself, to think about what's going on, about what I feel, about what I want to talk about and so on. This bit is certainly gone.
Extract 6. and that I miss this journey over, that it was some kind of me-time, when I go somewhere by public transport, I can indulge in my thoughts and sort it out.

In extract 5, the speaker positions the journey as an opportunity to re-consider things, what she wanted to talk about; in extract 6, the reference to ordering things also implies a finite process with a positive end. In both fragments, although implied, the journey not only serves a purpose, but it ends with a positive result.

Interestingly, the combination of the physical movement and reflection seems to be attractive even when psychotherapy had gone online. One of our informants talked about driving to the session and having the session in the car.

Extract 7. and I always get in the car, I plug my headphones into my phone or, I either drive somewhere quiet or I just stop somewhere out of the way. No, sometimes I happen to stop at a car park when I'm, like, I'm not going to make it to the session on time.

Notably, the fragment also shows the journey in agentive terms. Linguistically, the journey is a deliberate action.

In the next section, we take up the theme of replacing the actual journey to psychotherapy with a symbolic one. We wanted to explore the extent to which, in the informants' narratives, the physical journey to psychotherapy can be or is replaced by other time-extended activities.

### The Symbolic Journey

In this section we explore stories of activities whose function is to replace the physical journey to psychotherapy and the time for preparation it offered. Most our informants talked about taking the time to prepare for the session, although the stories differed significantly in what kind of activities were described. On the one hand, the informants talked about time-extended preparations of, for example, writing a diary as a way of preparing for the session, but on the other, they talked about thinking or making sure that their phone battery is fully loaded.

Our argument in this section is that in contrast to the stories of the physical journey, where the informants were constructing it without qualification in un-modalised verbs, here, the stories become more qualified. This is mostly done with the use of hedges (linguistic devices which introduce limits to the activity described, such as “usually,” “sometimes,” “kind of” and the like). The time before the session is constructed fraught with difficulties, as the informants often construct their actions as limited.

We start our discussion with the first group of abovementioned activities, those which take the most time. Consider:

Extract 8. I usually do it outside. There is this kind of a mini park, like, trees, a little bench and so on. To relax in a way during these conversations, as a talk with a psychologist is, you know, stressful on its own (…) I mean, I go to a place where I feel safe, to put it this way.
Extract 9. I keep a kind of a diary or a journal. And so, before a therapy session I try to recall what happened between the sessions, what was important to me, what I'd like to talk about.
Extract 10. Now I must make a real effort to actually find the time before the session and do a kind of recap, as there is a lot of things at home, like, okay, I'll check my email, this and that, so that's for sure.

In extract 8, even though the verbs the informant uses are un-modalized, the informant introduces a hedge at the beginning of the fragment. By inserting “usually”, the informant suggests that what she describes is not a matter of course, something that simply happens no matter what. Extract 9, in turn, contains a verbal limitation of the informant's activities, as she is constructed to “try” to do things.

The most limitations are conveyed in extract 10. First, the informant represents her activities through the mediation of the deontically (i.e., referring to obligation) modal “must” and thus, the activities are only an obligation, rather than an actuality. Moreover, the verb “must” is collocated with “make an effort” which linguistically removes the informant's activity even further.

Consider now the following extracts:

Extract 11. Ugh, as far as the technical (issues) are concerned, well, at this point we're already sort of online, on Skype, it's our second session, the one after we switched to Skype. When it comes to this, I try to wear, well, not necessarily pajamas - the ones you sometimes wear at home, but to get dressed as if I was walking into the (therapist's) office. I try to restart my computer before (the session) so that it's not, like, overloaded or something.
Extract 12. so now I kind of try to make sure that there are as few distractors or escapes as possible.
Extract 13. Yes, I always have the conversation from the dining (room). I sit on the sofa, there's nothing behind me to distract me. I also try not to leave too many things that would distract me on the table, that would make me want to grab them, play with them.

The three extracts above show similar constructions as discussed with reference to extracts 8–10. At least some activities are mediated through references to trying. We realize, of course, that there is no linguistic evidence as to why the informants use such hedging. It might, for example, result from the informants' unwillingness to claim success in the activities, rather than absence of time or the effort necessary to perform them.

Accepting this uncertainty, our main argument, however, is that the informants' stories about their physical journeys never included such linguistic devices. Physical journeys and the activities during them were consistently constructed at the highest level of linguistically constructed certainty. Consistently, there were no qualifications or hedging. Regardless of the source of the qualifications in the stories at hand, there is a contrast between the stories of the time before online psychotherapy sessions and those which happen in physical reality.

Now, in addition to stories which we have just discussed, there were a few examples in which preparations for a psychotherapy session at home were represented without qualifications, there were no linguistic hedges and the verbs were un-modalized. Consider:

Extract 14. I think about what I'm going to say, like, what I remembered most from this week, what kept me thinking. Or perhaps I remembered a dream that was important to me and (it was) difficult for me to interpret it on my own.
Extract 15. I think about what kind of problems I have, what I'd like to talk about, what subjects I'd actually like to discuss.
Extract 16. I prepare for the session in the same way (as before). 'Cause you might think that, I don't know, that you are online from the waist up and you can, I don't know, sit there wearing tracksuit bottoms. So, I can see that I prepare for it the same way as before (when it was) in the office. I wash my hair, I do my make-up, I get dressed as usual, just as if I were sitting there in her office.
Extract 17. I put a pillow under my back on the bed, I make sure that my phone is charged, 'cause I talk with my headphones on, so I don't have the possibility to talk and charge the headphones at the same time. So, I must remember about it.
Extract 18. As a matter of fact, before (the session) I turn my webcam on and check if you can even see me.

The first two extracts (14 and 15) refer to thinking as the time before a psychotherapy session. It is significant, however, that they are not contextualized in any way. The thinking is not constructed in time, either in terms of duration or positioning in relation to the session itself. The two references to thinking are unqualified, construct a response which offers a full commitment to its truth and certainty.

However, the lack of any social or temporal context, combined with the fact that the responses are almost generic in their content, may suggest that they are a way to circumvent the need for any detail. As they were elicited by questions about session preparation, we must wonder about the extent to which they are deliberately acontextual, only aimed to offer a positive response to a question from the interviewer.

Extract 16 on the other hand, focuses on personal grooming, which acknowledges the semi-public nature of psychotherapy. Here the informant offers more of a personal context to their response, explaining her wish to appear as like in the therapist's office. The response itself is again linguistically un-modalized, with no qualifications. This story is similar to those in Extracts 17 and 18, where the informants talk about a logistic aspect of preparations.

The key argument we would like to make is that the activities described in the three fragments are either usual everyday ones (such as personal grooming) or merely making sure that the session goes smoothly and comfortably. Nothing in those activities is constructed to replace the physical journey.

There are two ways in which the time before psychotherapy sessions is constructed in our informants' stories. On the one hand, there are explicit preparations activities. These, however, are described with hedging and other linguistic means distancing the informant from their unfettered agency. The activities which do not have such qualifications, on the other hand, are ones which are either ambiguous as to their time extension or, alternatively, are usual daily activities and logistic preparations for the session.

What our data suggest is that the physical journey that is consistently described in active, linguistically agentive terms, finds no replacement. The thinking and reflecting time before the session seems lost, or at least it does not find its way into the informants' stories.

Furthermore, this argument finds some support in the stories where our informants explicitly talk about missing the physical journey. In such stories, the journey is taken away from them, but nothing seems to replace it. In addition to the extracts we quoted above (e.g., extract 4), consider the following fragment:

Extract 20. And actually, I used to think about it right from the morning, that I was going to Justyna's (office) that day after work. So, it's like the whole day was this kind of very intensive day. Most of the time I try to pay attention to all my actions, needs, emotions and so on, but it used to be a whole day like this. When I was going to work and then I was going to Justyna's and then it was very intensive. And there's no such component here. I think that it also works slightly out of habit. For this (period of) time, for these two years, it's been kind of trained, kind of standard. And now it's not there. Although probably it's just a matter of time and of learning new mechanisms. Although I hope not and that we'll return to this face-to-face (psychotherapy).

The informant creates a contrast between the time she traveled to psychotherapy and one she does not. There is a clear shift between how the parts of the story are told. The first part is told constructing the informant in agentive terms (the only break in the agentivity is insertion of “try” with regard to paying attention the informant's behaviors). The second part of the story is different. The informant is no longer spoken about, she disappears from the story, so to say. In other words, there are no explicit references to the informant's activities, and the possibility of learning “new mechanisms” is not linguistically claimed by an individual. Such an impersonal construction is reinforced by the return of personal reference (the pronouns “we” referring presumably to herself and the therapist) when the informant ends with speaking about hope of returning to face-to-face therapy.

## Discussion

In this article we explore narrative experience of the journey to psychotherapy sessions and its absence after psychotherapy shifted to the online mode. Our main argument is that the physical journey is constructed not only as travel time, solitude which can be used for reflection, but, importantly, as an active process which ends with a resolution. Assumed in the literature to be a logistic and economical issue, we argue that journey to psychotherapy have meanings and is part of the therapeutic process.

Constructions of the physical journey in the informants' narratives are in contrast with those of the time before an online session. The constructions of unfettered agents engaging with the physical journey disappear and are replaced with stories of people whose actions are hedged and qualified. If there is clear agency, it is relegated to actions which are either generic or not immediately related to psychotherapy. Moreover, our data suggest the (narrated) experience of the physical journey seems not to be replaced (or, indeed, replaceable) by other actions. Time and again, we heard of a void, absence of physical journeys left in clients' lives.

As mentioned, the issue of the journey to a psychotherapy session has so far been seen mostly in terms of accessibility. The journey's absence is treated as an advantage of online therapy. Our research is closer to how Russell (2018) problematized the journey in her book “Screen relations”. She suggests that movement in space is key and helps experience the sense of the self. Whether the journey is to or from a session, it helps remembering the session. This is reflected in Essig and Russell's guidelines for clients in which they urge them to have a 15-min walk before and after a session (Essig and Russell, 2021). If walking is not an option, they suggest other forms of movement (e.g., stretching). Movement, they propose, helps internalizing the session. A similar perspective is taken by Janet Sayers (2021).

Russell's (2018) and Sayers (2021) perspectives are based on years-long therapy practice and conversations with clients and other therapists. In contrast, our article is supported by empirical data—interviews with clients of psychotherapeutic services who talked about changes to the communication channel resulting from the SARS-Cov2 pandemic. Text-based discourse analysis, which we adopt here, focuses its analytic efforts on exploring the objective lexico-grammatical structures of language. In the process, researchers' cultural backgrounds or life experiences are not interrogated in the replicable analysis texts' grammar and lexis. And as the lexico-grammatical form is largely uncontrolled by language users, such analysis offers a way in which to reveal how they construct their world.

What we would like to suggest, further, is that narratives of the physical journey to psychotherapy are usefully seen as constructing a rite of passage (van Gennep, 1960). van Gennep's (1960; see also Turner, 1969, 1974; for an overview see Hockey, 2002) initial observations and ways of accounting for cultural ways of shifting status, social position, or space resulted in the now classic phrase “rites of passage”, i.e., culturally sanctioned ways of transition between one state and another. As Leach (1964, 1976, 1977, 1982) points out, cultural categories used for the classification of the world form bi-polar oppositions: sacred-profane, up-down, one's own-stranger, inside-outside, and so on. But people move between the categories, they move in physical space (from outside inside, for example), they move from one status to another (as in acquiring priesthood, getting married or getting a doctorate), they move in social position (as in becoming monarchs, presidents or professors). Moments of transition, because they escape the duality, acquire a special, marked status—one which makes them sacred, dangerous, taboo (Eliade, 1966; also Douglas, 1984).

It is by now well-known in the social sciences of medicine that passage is also marked in medical settings. Births and deaths, motherhood, fatherhood, hospitalization to name a few of transition moments with which the medical profession must deal with and cope (for an overview see Helman, 2001, see also e.g., Murphy et al., 1988; Froggatt, 1997; Draper, 2003). The stories of the physical journey, we suggest, are stories of the passage between the non-therapy and therapy time. Their constructed importance in the stories of our informants also suggests that the time acquires meaning beyond the simple movement in time and space.

If our argument is plausible, it has a number of consequences especially for understanding the therapeutic process, which hitherto has been understood mostly in terms of the time and activities inside (in-session) and outside (inter-session) therapist's office (Orlinsky et al., 2004; Orlinsky, 2009; Hartmann et al., 2011). What we suggest here is that the dichotomy can be mediated by the time in-between, which is neither simply outside, nor inside, the physical journey to a psychotherapy session is the time of passing between the two states. It is a process of change, and not only a process of traveling.

We think that the journey should not be seen as part of the “inter-session” time. This is because it is explicitly devoted to a single upcoming session. It refers to a single journey to a single session and the context-bound, here-and-now passage from the time before to the time in the session, which the client actively takes.

## Limitations of the Study

There are some limitations of our study. On the one hand, qualitative research is not aimed at generalizations, on the other, a relatively small corpus, suggests caution in approaching our arguments. Yet, the consistency with which our informants talked about the journey to psychotherapy sessions and its absence, persuades us to make the arguments above, raising the questions both about the nature of the therapeutic process, and the role of the journey within it.

Our informant sample also has limitations. Our research was carried among persons who responded to an advertisement on internet fora for people using psychotherapeutic services. Using such a recruitment strategy carries the risk of self-selection bias. We chose the strategy as, given the timescale of the project, other strategies were not available. We recognize that recruitment through psychotherapists could result in a more diverse sample. However, it must be noted that such alternative recruitment procedures give rise to a number of ethical issues.

We acknowledge that the particularity of the timing of the project, as it was carried out 2 months after the introduction of a lockdown in Poland. The lockdown was unexpected and made an abrupt change to people's lives and is likely to have resulted in feeling overwhelmed or disoriented. This might be seen as significant for the collection of our data, and in particular the sources underpinning the narratives we gathered.

It must be noted, however, that people's stories are always socially situated and made in a multitude of contexts. The full national lockdown is likely to have created multiple contexts for our informants, all of which could have had a significant impact on how they talked. Some of those contexts will never be explored or, indeed, even identified.

It is also important to stress that acknowledging the existence of such contexts does not invalidate the results of our study. Rather, it contextualizes them, while future research can shed further light on them. Moreover, we were mostly interested in the linguistic form of the stories. People do not control it and while the lockdown is likely to have had impact on what people said, it is extremely unlikely to have had any impact on how people have said it.

Furthermore, absence of earlier research on narrative experience of the absence of the physical journey to psychotherapy sessions makes interpretation of our study more difficult. We postulate therefore further exploration of meanings and roles of the physical journey to psychotherapy both in narratives of clients and therapists. Here are, we think, some of the important questions which should be asked in further research. How important is the journey? How significant is its absence? What is its position and role within the therapeutic process? Can it be replaced by other activities? For what kind of clients is important, what is its impact on the relationship with the therapist? The questions are particularly important given the globally occurring changes to practicing psychotherapy as well as more and more common shift to practicing it online.

## Data Availability Statement

The raw data supporting the conclusions of this article will be made available by the authors, without undue reservation.

## Ethics Statement

The studies involving human participants were reviewed and approved by Ethics Committee of the Wroclaw Faculty of the University of Social Sciences and Humanities. The patients/participants provided their written informed consent to participate in this study.

## Author Contributions

JZ and DG contributed to conception and design of the study, performed data analysis, and coded the data. JZ organized the dataset. DG wrote the first draft. MW wrote sections of the manuscript and performed literature searches. All authors contributed to manuscript revision, read, and approved the submitted version.

## Conflict of Interest

The authors declare that the research was conducted in the absence of any commercial or financial relationships that could be construed as a potential conflict of interest.

## Publisher's Note

All claims expressed in this article are solely those of the authors and do not necessarily represent those of their affiliated organizations, or those of the publisher, the editors and the reviewers. Any product that may be evaluated in this article, or claim that may be made by its manufacturer, is not guaranteed or endorsed by the publisher.

## References

[B1] Ashwick R. Turgoose D. Murphy D. (2019). Exploring the acceptability of delivering Cognitive Processing Therapy (CPT) to UK veterans with PTSD over Skype: a qualitative study. Eur. J. Psychotraumatol. 10, 1573128. 10.1080/20008198.2019.1573128 30774784 PMC6366402

[B2] Barker C. Galasiński D. (2001). Cultural Studies and Discourse Analysis: A Dialogue on Language and Identity. London; Thousand Oaks, CA: Sage

[B3] Berryhill M. Culmer N. Williams N. Halli-Tierney A. Betancourt A. Roberts H.. (2018). Videoconferencing psychotherapy and depression: a systematic review. Telemed. E-Health 25, 58. 10.1089/tmj.2018.0058 30048211

[B4] Biancalani G. Franco C. Guglielmin M. S. Moretto L. Orkibi H. Keisari S.. (2021). Tele-psychodrama therapy during the COVID-19 pandemic: participants' experiences. Arts Psychother. 75, 101836. 10.1016/j.aip.2021.101836 34305221 PMC8294105

[B5] Botta A. A. Holmes-Maxwell T. Williams C. R. (2021). Reflections on virtual group work with transgender and gender diverse youth during the pandemic. Soc. Work Groups 44, 111–116. 10.1080/01609513.2020.1868693

[B6] Braun V. Clarke V. (2006). Using thematic analysis in psychology. Qualitative Research in Psychology, 3, 77–10. http://dx. 10.1191/1478088706qp063oa32100154

[B7] Campbell A. M. (2020). An increasing risk of family violence during the Covid-19 pandemic: strengthening community collaborations to save lives. Foren. Sci. Int. Rep. 2, 100089. 10.1016/j.fsir.2020.100089 PMC7152912 38620174

[B8] Chen C. K. Nehrig N. Wash L. Schneider J. A. Ashkenazi S. Cairo E.. (2020). When distance brings us closer: Leveraging tele-psychotherapy to build deeper connection. Counsel. Psychol. Q. 34, 1–14. 10.1080/09515070.2020.1779031

[B9] Douglas M. (1984). Purity and Danger: An Analysis of Concepts of Pollution and Taboo. London: Routledge.

[B10] Draper J. (2003). Men's passage to fatherhood: an analysis of the contemporary relevance of transition theory. Nursing Inquiry 10, 66–78. 10.1046/j.1440-1800.2003.00157.x 12622806

[B11] Eliade M. (1966). Traktat o historii religii (Polish translation of: Traite d'historie des religions). Warszawa, Poland: Ksiazka i Wiedza.

[B12] Essig T Russell G.I. (2021). Remote Session Guidelines for Periods of Restricted Travel. Available online at: https://apsa.org/sites/default/files/Guide3-24.pdf

[B13] Fairclough N. (1992). Discourse and Social Change. Cambridge: Polity Press.

[B14] Fairclough N. (1995). Critical Discourse Analysis. London: Longman.

[B15] Fairclough N. (2003). Analysing Discourse. London: Routledge.

[B16] Fleuty K. Almond M. K. (2020). Remote access therapy for veterans with psychological problems: current state of the art. Milit. Med. 185, e1046–e1050. 10.1093/milmed/usaa020 32060551

[B17] Froggatt K. (1997). Rites of passage and the hospice culture. Mortality 2, 123–136. 10.1080/713685862

[B18] Glueckauf R. L. Maheu M. M. Drude K. P. Wells B. A. Wang Y. Gustafson D. J.. (2018). Survey of psychologists' telebehavioral health practices: technology use, ethical issues, and training needs. Prof. Psychol. Res. Pract. 49, 205–219. 10.1037/pro0000188

[B19] Guinart D. Marcy P. Hauser M. Dwyer M. Kane J. M. (2020). Patient attitudes toward telepsychiatry during the COVID-19 pandemic: a nationwide, multisite survey. JMIR Mental Health 7, e24761. 10.2196/24761 33302254 PMC7758084

[B20] Halliday M. A. K. (1978). Language and Social Semiotic. London: Edward Arnold.

[B21] Halliday M. A. K. (1994). An Introduction to Functional Grammar, 2nd Edn. London: Edward Arnold.

[B22] Halliday M. A. K. Hasan R. (1985). Language, Context and Text: Aspects of Language in a Social-Semiotic Perspective. Geelong: Deakin University Press.

[B23] Hartmann A. Orlinsky D. Zeeck A. (2011). The structure of intersession experience in psychotherapy and its relation to the therapeutic alliance. J. Clin. Psychol. 67, 1044–1063. 10.1002/jclp.20826 21800294

[B24] Helman C. (2001). Culture, Health and Illness, 4th Edn. London: Arnold.

[B25] Hockey J. (2002). The importance of being intuitive: Arnold Van Gennep's the rites of passage. Mortality 7, 210–217. 10.1080/135762702317447768

[B26] Hodge R. I. V. Kress G. R. (1993). Language as Ideology. London: Routledge.

[B27] Humer E. Pieh C. Kuska M. Barke A. Doering B. K. Gossmann K.. (2020). Provision of psychotherapy during the COVID-19 pandemic among Czech, German and Slovak Psychotherapists. Int. J. Environ. Res. Public Health 17, 4811. 10.3390/ijerph17134811 32635422 PMC7370023

[B28] Jobes D. A. Crumlish J. A. Evans A. D. (2020). The COVID-19 pandemic and treating suicidal risk: the telepsychotherapy use of CAMS. J. Psychother. Integ. 30, 226–237. 10.1037/int0000208

[B29] Khanra S. Mukherjee A. Goyal N. Das B. Munda S. K. (2021). Service utilization and saved travel cost in telepsychiatry consultation by outpatients at a psychiatric hospital in India during COVID-19 pandemic. Asian J. Psychiatry 57, 102568. 10.1016/j.ajp.2021.102568 33535135 PMC7840431

[B30] Khazaie H. Rezaie L. Shahdipour N. Weaver P. (2016). Exploration of the reasons for dropping out of psychotherapy: a qualitative study. Eval. Prog. Plan. 56, 23–30. 10.1016/j.evalprogplan.2016.03.002 27010417

[B31] Kopelovich S. L. Turkington D. (2021). Remote CBT for psychosis during the COVID-19 pandemic: challenges and opportunities. Comm. Mental Health J. 57, 30–34. 10.1007/s10597-020-00718-0 33001323 PMC7528451

[B32] Kvale S. Brinkmann S. (2009). Interviews: Learning the Craft of Qualitative Research Interviewing. London: Sage.

[B33] Leach E. R. (1964). “Anthropological aspects of language: animal categories and verbal abuse,” in New Directions in the Study of Language, eds. Lenneberg E. H. (Cambridge, Mass: MIT Press), 23–63.

[B34] Leach E. R. (1976). Culture and Communication: The Logic by Which Symbols are Connected—An Introduction to the Use of Structuralist Analysis in Social Anthropology. Cambridge: Cambridge University Press.

[B35] Leach E. R. (1977). Custom, Law, and Terrorist Violence. Edinburgh: Edinburgh University Press.

[B36] Leach E. R. (1982). Social Anthropology. Oxford: Oxford University Press.

[B37] Miles M. B. Huberman A. M. (1994). Qualitative Data Analysis: An Expanded Sourcebook. London: Sage.

[B38] Miu A. S. Vo H. T. Palka J. M. Glowacki C. R. Robinson R. J. (2020). Teletherapy with serious mental illness populations during COVID-19: telehealth conversion and engagement. Couns. Psychol. Q. 34, 1–18. 10.1080/09515070.2020.1791800

[B39] Murphy R. F. Scheer J. Murphy Y. Mack R. (1988). Physical disability and social liminality: a study in the rituals of adversity. Soc. Sci. Med. 26, 235–242. 10.1016/0277-9536(88)90244-4 2964721

[B40] Norman S. (2006). The use of telemedicine in psychiatry. J. Psychiatr. Mental Health Nurs. 13, 771–777. 10.1111/j.1365-2850.2006.01033.x 17087682

[B41] Norwood C. Moghaddam N. G. Malins S. Sabin-Farrell R. (2018). Working alliance and outcome effectiveness in videoconferencing psychotherapy: a systematic review and noninferiority meta-analysis. Clin. Psychol. Psychother. 25, 797–808. 10.1002/cpp.2315 30014606

[B42] Orlinsky D. E. (2009). The “generic model of psychotherapy” after 25 years: evolution of a research-based metatheory. J. Psychother. Integ. 19, 319–339. 10.1037/a0017973

[B43] Orlinsky D. E. Rønnestad M. H. Willutzki U. (2004). “Fifty years of psychotherapy process-outcome research: Continuity and change,” in Bergin and Garfield's Handbook of Psychotherapy and Behavior Change, 5th Edn. eds. Lambert M. (New York, NY: Wiley), 307–389.

[B44] Potter J. Wetherell M. (1987). Discourse and Social Psychology. London: Sage Publications.

[B45] Russell G. I. (2018). Screen Relations: The Limits of Computer-Mediated Psychoanalysis and Psychotherapy, 1st Edn. London: Routledge.

[B46] Sayers J. (2021). Online psychotherapy: transference and countertransference issues. Br. J. Psychother. 37, 223–233. 10.1111/bjp.12624

[B47] Titscher S. Meyer M. Wodak R. Vetter E. (2000). Methods of text and discourse analysis. London: Sage.

[B48] Turgoose D. Murphy D. Ashwick R. (2018). Exploring the feasibility and acceptability of using tele-therapy for UK veterans with PTSD. Final Report. Combat Stress and Forces in Mind Trust.

[B49] Turner V. W. (1969). The Ritual Process: Structure and Anti-Structure. Chicago: Aldine.

[B50] Turner V. W. (1974). Dramas, Fields, and Metaphors: Symbolic Action in Human Society. Ithaca: Cornell University Press.

[B51] van Gennep A. (1960). The Rites of Passage. (Translated by Monika B. Vizedom and Gabrielle L. Caffee). Chicago: The University of Chicago Press. [First published in 1908 as Les rites de passage, Paris: E, Guilmote.]

[B52] Van Leeuwen T. Wodak R. (1999). Legitimizing immigration control: a discourse-historical analysis. Discourse Studies 1(1):83–118. 10.1177/1461445699001001005

[B53] Wang C. Pan R. Wan X. Tan Y. Xu L. Ho C. S.. (2020). Immediate psychological responses and associated factors during the initial stage of the 2019 coronavirus disease (COVID-19) epidemic among the general population in China. Int. J. Environ. Res. Public Health 17, E1729. 10.3390/ijerph17051729 32155789 PMC7084952

[B54] Wittson C. L. Affleck D. C. Van J. (1961). Two-way television in group therapy. Psychiatr. Serv. 12, 22–23. 10.1176/ps.12.11.22 14007789

